# PUNCH CD3-OLS: A Phase 3 Prospective Observational Cohort Study to Evaluate the Safety and Efficacy of Fecal Microbiota, Live-jslm (REBYOTA) in Adults With Recurrent *Clostridioides difficile* Infection

**DOI:** 10.1093/cid/ciae437

**Published:** 2024-08-24

**Authors:** Paul Feuerstadt, Teena Chopra, Whitfield Knapple, Nicholas W Van Hise, Erik R Dubberke, Brian Baggott, Beth Guthmueller, Lindy Bancke, Michael Gamborg, Theodore S Steiner, Daniel Van Handel, Sahil Khanna

**Affiliations:** Digestive Diseases, Yale School of Medicine, New Haven, Connecticut, USA; Division of Infectious Diseases, Wayne State University, Detroit, Michigan, USA; Arkansas Gastroenterology, North Little Rock, Arkansas, USA; Metro Infectious Disease Consultants, Burr Ridge, Illinois, USA; Division of Infectious Diseases, Washington University School of Medicine, Saint Louis, Missouri, USA; Department of Gastroenterology, Hepatology and Nutrition, Cleveland Clinic, Cleveland, Ohio, USA; Global Clinical and Translational Sciences, Ferring Pharmaceuticals, Roseville, Minnesota, USA; Global Clinical and Translational Sciences, Ferring Pharmaceuticals, Roseville, Minnesota, USA; Global Clinical Operations US, Ferring Pharmaceuticals, Roseville, Minnesota, USA; Global Biometrics, Ferring Pharmaceuticals, Copenhagen, Denmark; Division of Infectious Diseases, University of British Columbia, Vancouver, Canada; MNGI Digestive Health, Plymouth, Minnesota, USA; Department of Gastroenterology and Hepatology, Mayo Clinic, Rochester, Minnesota, USA

**Keywords:** CDI, *Clostridioides difficile*, dysbiosis, live biotherapeutic product, RBX2660

## Abstract

**Background:**

The aim of this study was to evaluate the safety and efficacy of fecal microbiota, live-jslm (RBL; REBYOTA)—the first single-dose, broad consortia microbiota-based live biotherapeutic approved by the US Food and Drug Administration for preventing recurrent *Clostridioides difficile* infection (rCDI) in adults following standard-of-care (SOC) antibiotic treatment.

**Methods:**

PUNCH CD3-OLS was a prospective, phase 3, open-label study, conducted across the US and Canada. Participants were aged ≥18 years with documented rCDI and confirmed use of SOC antibiotics. Participants with comorbidities including inflammatory bowel disease and mild-to-moderate immunocompromising conditions could be enrolled. A single dose of RBL was rectally administered within 24–72 hours of antibiotic completion. The primary endpoint was the number of participants with RBL- or administration-related treatment-emergent adverse events (TEAEs). Secondary endpoints included treatment success and sustained clinical response, at 8 weeks and 6 months after RBL administration, respectively.

**Results:**

Overall, 793 participants were enrolled, of whom 697 received RBL. TEAEs through 8 weeks after administration were reported by 47.3% of participants; most events were mild or moderate gastrointestinal disorders. Serious TEAEs were reported by 3.9% of participants. The treatment success rate at 8 weeks was 73.8%; in participants who achieved treatment success, the sustained clinical response rate at 6 months was 91.0%. Safety and efficacy rates were similar across demographic and baseline characteristic subgroups.

**Conclusions:**

RBL was safe and efficacious in participants with rCDI and common comorbidities. This is the largest microbiota-based live biotherapeutic study to date, and findings support use of RBL to prevent rCDI in a broad patient population.

**Clinical Trials Registration:**

NCT03931941.

## Teaser

In this prospective, phase 3, open-label study, that fecal microbiota, live-jslm (RBL) was safe and efficacious for prevention of recurrent *Clostridioides difficile* infection in a broad patient population inclusive of those with inflammatory bowel disease and mild-to-moderate immunocompromising conditions.

## Graphical Abstract

**Graphical Abstract:**
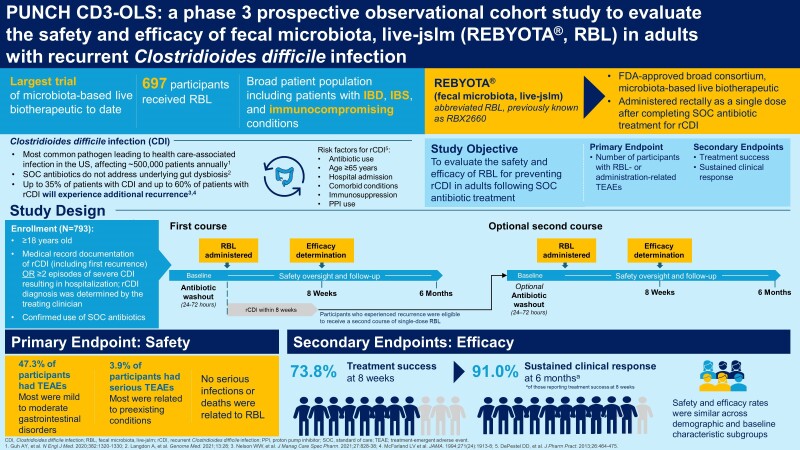

*Clostridioides difficile* infection (CDI) is the leading cause of healthcare-associated infections in the United States (US) [1], with a significant clinical and socioeconomic burden [2, 3]. Risk factors for recurrent CDI (rCDI) include antibiotic use, age ≥65 years, hospital admission, comorbid conditions (eg, chronic kidney disease, inflammatory bowel disease [IBD]), immunosuppression, and history of CDI [4, 5]. Index CDI and recurrent episodes are treated with standard-of-care (SOC) antibiotics, which treat the vegetative phase of the infection. However, these agents do not address the underlying dysbiosis associated with recurrence. A healthy, diverse microbiota can control the spore phase, but remnant dysbiosis after SOC antimicrobials can leave patients at an elevated risk of rCDI via conversion of the spore to the vegetative phase [6]. Microbiota-based therapies have been shown to decrease rCDI frequency [7, 8].

Fecal microbiota, live-jslm (REBYOTA, abbreviated here as RBL, previously known as RBX2660) is the first US Food and Drug Administration (FDA)–approved single-dose, broad consortium, microbiota–based live biotherapeutic indicated for rCDI prevention in adults following SOC antibiotic treatment for rCDI [9]. FDA approval was based on RBL efficacy evaluated in the multicenter, prospective, randomized, double–blind, placebo-controlled phase 3 trial (PUNCH CD3; NCT03244644). Using a Bayesian primary analysis integrating data from a placebo-controlled phase 2 trial, the estimated rate of treatment success was significantly higher in the RBL group (70.6%) than in the placebo group (57.5%), with a 99.1% posterior probability of superiority [8]. RBL was also found to be safe, with most of the reported adverse events (AEs) following administration being mild-to-moderate gastrointestinal disorders including diarrhea, abdominal pain, and nausea.

While the safety and efficacy of some live microbiota-based therapies, such as RBL, have been demonstrated in randomized controlled trials (RCTs), these studies commonly excluded participants with comorbidities encountered in real-world clinical settings. PUNCH CD3–OLS is the largest study of a live microbiota-based therapy conducted to date and included participants with comorbidities such as IBD, irritable bowel syndrome (IBS), and mild-to-moderate immunocompromising conditions. The final safety and efficacy findings from this phase 3, open-label study (PUNCH CD3–OLS; NCT03931941) are reported here.

## METHODS

PUNCH CD3-OLS was a prospective, phase 3, open-label study conducted across the US and Canada under a US FDA Investigational New Drug Application and a Health Canada Clinical Trial Application. Each participating site obtained approval of the protocol from an institutional review board/research ethics board. The study was conducted in accordance with the ethical standards of the 1964 Declaration of Helsinki. Participants provided written informed consent to participate prior to initiation of any study procedures.

### Study Population

The study population included participants ≥18 years of age with a current or past diagnosis of rCDI as determined by the treating physician, or ≥2 episodes of severe CDI resulting in hospitalization, as these patients may be at risk of recurrence. rCDI diagnosis methods were not dictated and the treating clinician could use methods customary at the study site. Those with diarrhea caused by a confirmed intestinal pathogen other than *C. difficile* were excluded from the study per the exclusion criteria. Other study details, including full inclusion and exclusion criteria, are shown in the Supplementary Methods.

### RBL Administration

RBL [9] was rectally administered as a single 150-mL dose 24–72 hours after completing CDI antibiotic therapy, without bowel preparation. Participants were eligible to receive a second course of single–dose RBL within 21 calendar days if they met the criteria for treatment failure. Treatment failure was defined as the presence of CDI diarrhea within 8 weeks of RBL administration. Recurrence was confirmed by a positive stool test (C. DIFF QUIK CHEK COMPLETE Test) for *C. difficile* glutamate dehydrogenase antigen and toxins A and B as determined by the central laboratory. Use of antibiotics prior to a second course of RBL was at the discretion of the investigator. If antibiotics were given to control symptoms, a 24- to 72-hour washout period was required prior to administration of RBL.

### Study Outcomes

The primary endpoint was the number of participants with RBL- or administration-related treatment-emergent adverse events (TEAEs). TEAEs were defined as any AE occurring on or after the day of first RBL administration and were coded using the Medical Dictionary for Regulatory Activities (MedDRA), version 20.0. Safety data were censored at CDI recurrence. The full definition of serious TEAEs is provided in the Supplementary Methods. TEAEs are presented by MedDRA system organ classes (type) and preferred terms (exact description of medical condition). TEAEs were collected using participant diaries (via solicited events recorded for week 1), in-office follow-up visits (weeks 1 and 8), and telephone assessments (week 4, months 4 and 6). Supportive safety endpoints included severity, relatedness, and select CDI-related TEAEs through 8 weeks after RBL administration. A medical monitor reviewed all AEs and serious TEAEs for potential safety trends. Reviews were presented to the data and safety monitoring board for analysis and adjudication of potential stopping rules.

Secondary endpoints included treatment success and sustained clinical response. Treatment success was defined as the absence of CDI diarrhea through 8 weeks after RBL administration. Sustained clinical response was defined as treatment success of the presenting CDI episode and no new CDI episodes through 6 months after RBL administration. Efficacy outcomes were initially determined by the site investigator, with subsequent independent adjudication by the Endpoint Adjudication Committee. Further details on the secondary endpoints are in the Supplementary Methods.

### Statistical Analysis

Descriptive statistics were reported for all endpoints. Treatment success and sustained clinical response (dependent variables) were summarized according to baseline population characteristics using univariate logistic regression analysis. Independent variables included age, sex, race, ethnicity, site geography, number of previous CDI episodes at baseline, and Charlson Comorbidity Index (CCI) score at baseline. Multivariate regression analysis methods and definitions of the study populations can be found in the Supplementary Methods.

## RESULTS

### Participants

**Figure 1.**
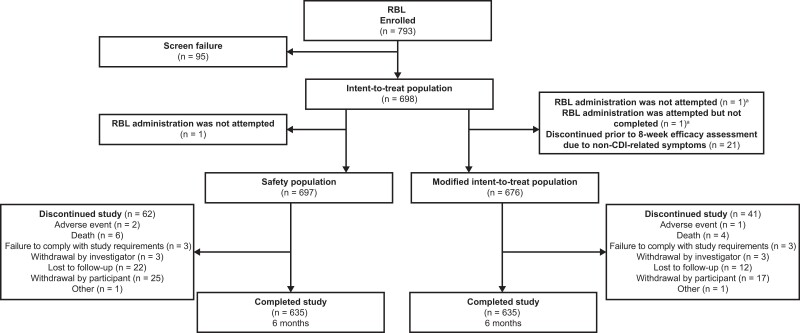
Participant flow diagram showing the enrollment, fecal microbiota, live-jslm (RBL) administration, and follow-up process for PUNCH CD3-OLS. ^a^RBL administration was attempted but not completed in 1 participant who discontinued prior to the 8-week efficacy assessment; this participant was counted in both categories shown. Abbreviation: CDI, *Clostridioides difficile*.

A total of 793 participants were enrolled between July 2019 and December 2022 in the US and Canada; the study was completed in July 2023. Ninety-five participants did not meet the study eligibility criteria, and 1 participant was enrolled but did not receive RBL. Overall, 697 participants and 676 participants were included in the safety (ie, received RBL) and modified intent-to-treat (mITT) populations, respectively (Figure 1). A total of 635 participants completed the study (Supplementary Table 1).

Most participants were White (93.8%) and female (69.9%) (Table 1). A total of 141 (20.2%) participants were immunocompromised due to immunocompromising conditions and/or medications. The enrolling CDI episode was diagnosed via polymerase chain reaction (PCR)–based testing for 62.0% of participants. Vancomycin was the most used antibiotic for the enrolling CDI episode (84.6%). A total of 26.7% of participants received RBL for their first recurrence (ie, second CDI episode), while 38.5% and 34.3% had experienced 3 or ≥4 prior episodes (inclusive of the enrolling episode), respectively. Documented medical history included gastrointestinal disorders (Crohn‘s disease [CD], n = 25; ulcerative colitis [UC], n = 45; unspecified IBD, n = 4; IBS, n = 97; gastroesophageal reflex disease, n = 288); chronic kidney disease, n = 74; and immunocompromising conditions and medications (malignant tumors, n = 42; other medical history [including end-stage renal disease, renal failure, and human immunodeficiency virus], n = 19; concomitant corticosteroids, n = 23; concomitant noncorticosteroid immunosuppressants, n = 92) (Table 1). One patient each had cirrhosis or was receiving peritoneal dialysis.

**Table 1.** Baseline Demographics, Characteristics, and Disposition (Safety Population)

Characteristic	RBL (N = 697)
Age ≥65 y	338 (48.5)
Sex, female	487 (69.9)
Race, White	654 (93.8)
No. of previous CDI episodes^a,b^	
2	186 (26.7)
3	268 (38.5)
≥4	239 (34.3)
Enrolling diagnostic test^c^	
PCR	432 (62.0)
EIA	75 (10.8)
GDH	50 (7.2)
Other^d^	65 (9.3)
Most recent antibiotic received	
Vancomycin	590 (84.6)
Fidaxomicin	99 (14.2)
Rifaximin	7 (1.0)
Charlson Comorbidity Index	
<3	329 (47.2)
≥3	368 (52.8)
GI comorbidities	
Ulcerative colitis	45 (6.5)
Crohn‘s disease	25 (3.6)
IBD (unspecified)^e^	4 (0.6)
IBS	97 (13.9)
GERD	288 (41.3)
Renal and urinary comorbidities	
CKD	74 (10.6)
Chronic urinary tract infections	61 (8.8)
Mild-to-moderate immunocompromising conditions^f^	
Malignant tumors	42 (6.0)
Other medical history^g^	19 (2.7)
Concomitant immunocompromising medications^f,h^	
Corticosteroids	23 (3.3)
Noncorticosteroids	92 (13.2)
RBL courses received	
Received 1 course of RBL	697 (100.0)
Received 2 courses of RBL	121 (17.4)

Data are presented as No. (%) of participants.

Abbreviations: CDI, *Clostridioides difficile* infection, CKD, chronic kidney disease; EIA, enzyme immunoassay; GDH, glutamate dehydrogenase; GERD, gastroesophageal reflex disease; GI, gastrointestinal; IBD, inflammatory bowel disease; IBS, irritable bowel syndrome; PCR, polymerase chain reaction; RBL, fecal microbiota, live-jslm.

^a^Inclusive of the enrolling episode.

^b^Number of CDI episodes recorded was incomplete for 4 participants.

^c^Enrolling tests could be used in combination.

^d^Category other for enrolling diagnostic tests included medical record documentation as toxins A and B, loop-mediated isothermal amplification.

^e^Unspecified IBD subgroup includes participants with medical history of inflammatory bowel disease.

^f^Participants were considered immunocompromised if they had a medical history of an immunocompromising condition and/or immunocompromising medication (ie, participants may meet >1 condition).

^g^Immunocompromising conditions were identified by searching the following terms in medical history: end-stage renal disease, renal failure, asplenia, human immunodeficiency virus (HIV) or HIV infection, congenital hemoglobinopathies, and immunodeficiency syndromes.

^h^Immunocompromising medications included corticosteroids and systemic immunosuppressive medications.

### Safety

A total of 398 participants experienced at least 1 TEAE throughout the entire study period. Most TEAEs were reported within 8 weeks of RBL administration and decreased thereafter. TEAEs were reported by 47.3% of participants within 8 weeks of RBL administration and by 23.0% of participants between 8 weeks and 6 months of follow-up (Table 2). Most TEAEs were mild or moderate in severity. The number of TEAEs were comparable across baseline characteristics (Supplementary Table 2).

**Table 2.** Summary of Treatment-Emergent Adverse Events Within 8 Weeks and Between 8 Weeks and 6 Months of Follow-up of Fecal Microbiota, Live-jslm Administration (Safety Population)

Adverse Event	Within 8 Weeks (N = 697)	8 Weeks to 6 Months (N = 697)
	Events/Participants (% of Participants)	
All TEAEs	794/330 (47.3)	331/160 (23.0)
TEAEs by maximum severity^a^		
Mild	456/142 (20.4)	141/51 (7.3)
Moderate	261/138 (19.8)	143/79 (11.3)
Severe	73/46 (6.6)	41/27 (3.9)
Potentially life-threatening	4/4 (0.6)	4/3 (0.4)
All serious TEAEs	35/27 (3.9)	37/25 (3.6)
Serious TEAEs by maximum severity^a^		
Mild	1/1 (0.1)	1/1 (0.1)
Moderate	8/8 (1.1)	2/2 (0.3)
Severe	14/14 (2.0)	19/19 (2.7)
Potentially life-threatening	4/4 (0.6)	3/3 (0.4)
Serious TEAEs by relatedness^b^		
Related to RBL	1/1 (0.1)^c^	0/0 (0.0)
Related to administration procedure	0/0 (0.0)	0/0 (0.0)
Related to CDI	1/1 (0.1)	1/1 (0.1)
Related to preexisting conditions	23/19 (2.7)	25/18 (2.6)
TEAEs leading to withdrawal from study	4/4 (0.6)	1/1 (0.1)
TEAEs leading to death^d^	3/3 (0.4)	1/1 (0.1)

Abbreviations: CDI, *Clostridioides difficile* infection; RBL, fecal microbiota, live-jslm; TEAE, treatment-emergent adverse event.

^a^Both participants and events are by maximum severity per participant.

^b^Relatedness categories are not mutually exclusive.

^c^Ulcerative colitis (n = 1).

^d^Deaths were due to cardiac arrest, cardiac failure, spina bifida, and pulmonary sepsis (all n = 1). All deaths were assessed as unrelated to RBL.

**Figure 2.**
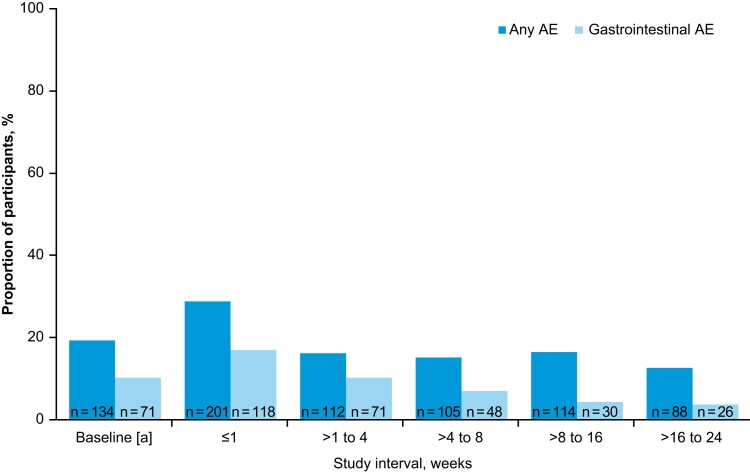
Summary of adverse events (AEs) by time interval (safety population). ^a^Baseline is defined as an AE onset date prior to fecal microbiota, live-jslm administration.

The most common TEAEs throughout the study period were gastrointestinal disorders reported by 29.4% of participants within 8 weeks of RBL administration and 7.3% of participants between 8 weeks and 6 months (Table 3). Gastrointestinal TEAEs were most commonly reported during the first week after RBL administration and steadily decreased thereafter (Figure 2).

**Table 3.** Treatment-Emergent Adverse Events by System Organ Class and Preferred Term in ≥5% of Participants Within 8 Weeks and Between 8 Weeks and 6 Months of Follow-up of Fecal Microbiota, Live-jslm Administration (Safety Population)

Adverse Event	Within 8 Weeks (N = 697)	8 Weeks to 6 Months (N = 697)
	Events/Participants (% of Participants)	
Gastrointestinal disorders	411/205 (29.4)	82/51 (7.3)
Diarrhea	95/85 (12.2)	21/19 (2.7)
Abdominal pain	78/70 (10.0)	10/10 (1.4)
Nausea	44/41 (5.9)	5/5 (0.7)
Abdominal distension	46/38 (5.5)	2/2 (0.3)
Infections^a^	78/71 (10.2)	86/68 (9.8)
General disorders^b^	44/36 (5.2)	10/8 (1.1)
Investigations^c^	49/35 (5.0)	5/5 (0.7)

Coding was based on Medical Dictionary for Regulatory Activities, version 20.0.

^a^The most commonly reported infections within 8 weeks were urinary tract infection (n = 8), coronavirus infection (n = 6), pneumonia (n = 4), and vulvovaginal mycotic infection (n = 4). Between 8 weeks and 6 months, the most commonly reported infections were urinary tract infection (n = 16) and coronavirus infection (n = 15).

^b^The most commonly reported general disorders within 8 weeks were chills (n = 14), fatigue (n = 12), and pyrexia (n = 9). Between 8 weeks and 6 months, the most commonly reported general disorders were noncardiac chest pain (n = 3) and pyrexia (n = 2).

^c^The most commonly reported investigations within 8 weeks were diastolic blood pressure decrease (n = 14) and systolic blood pressure increase (n = 4). Between 8 weeks and 6 months, all investigations were reported by 1 participant (blood potassium decreased, colonoscopy, coronavirus test positive, heart rate increased, and weight decreased).

Serious TEAEs were reported by 3.9% of participants within 8 weeks of RBL administration (Table 2). Most serious TEAEs were related to preexisting conditions. One participant experienced a serious TEAE of a UC flare; this event was assessed as being definitely related to a preexisting condition given the participant's underlying diagnosis of severe UC and assessed as possibly related to RBL. Serious TEAEs were reported in 3.6% of participants between 8 weeks and 6 months of follow-up. All serious infections that occurred during the study period were assessed as unrelated to RBL or its administration. Four participants experienced complications related to CDI recurrence within 8 weeks of RBL administration. None were assessed as related to RBL; these complications included septic shock (n = 4), intensive care unit admission (n = 2), and death (n = 1) (Supplementary Table 3). No CDI-related complications occurred within the 8-week to 6-month follow-up. No participant experienced toxic megacolon, colonic perforation, or emergency colectomy and no systemic infections were considered related to RBL or its administration. Four deaths, all of which were deemed unrelated to RBL, occurred within 8 weeks of RBL administration (n = 3) and between 8 weeks and 6 months of follow-up (n = 1) (Table 2).

### Efficacy

**Figure 3.**
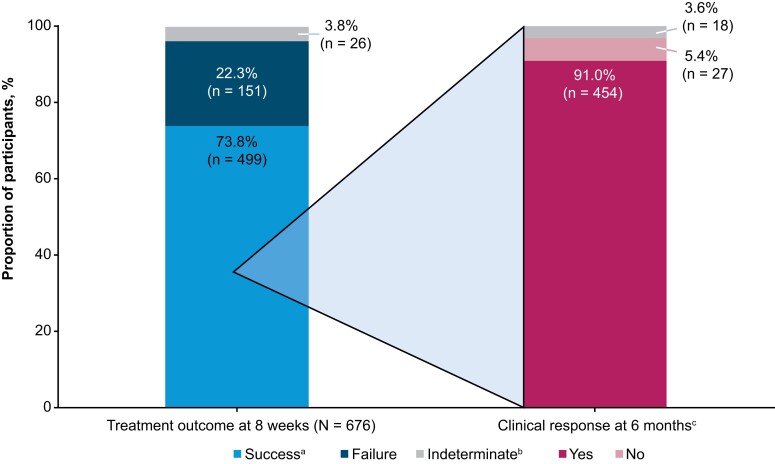
Efficacy outcomes within 8 weeks of fecal microbiota, live-jslm (RBL) administration and sustained clinical response at 6 months in 8-week treatment responders (modified intent-to-treat population). ^a^Treatment success is defined as the absence of *Clostridioides difficile* infection (CDI) diarrhea through 8 weeks after RBL administration. ^b^Efficacy outcome was indeterminate if CDI test was inconclusive or missed at time of visit. ^c^Sustained clinical response rate is defined as treatment success of the presenting CDI recurrence and no new CDI episodes through 6 months after RBL administration.

**Figure 4.**
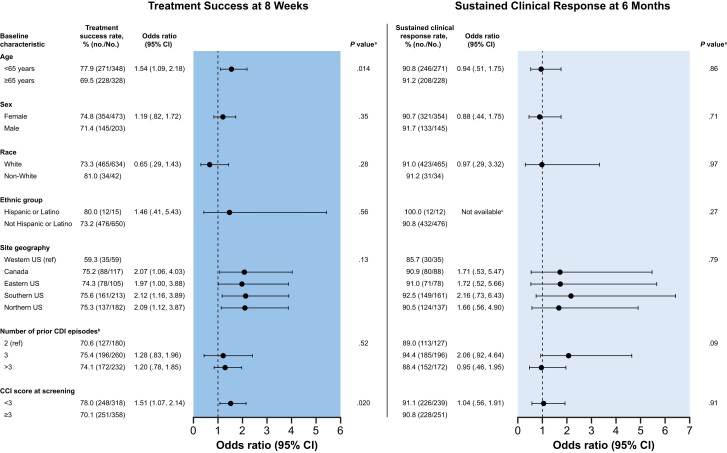
Subgroup analysis of treatment success rates at 8 weeks and sustained clinical response through 6 months across participant baseline characteristics (modified intent-to-treat population). ^a^*P* values were obtained from a univariate χ^2^ test. ^b^Participants with incomplete records for the number of *Clostridioides difficile* infection (CDI) events were excluded from the analysis (n = 4). ^c^Odds ratio not available, as none of the 12 Hispanic or Latino participants with 8-week treatment success had a new CDI episode between 8 weeks and 6 months. Abbreviations: CCI, Charlson Comorbidity Index; CI, confidence interval; ref, reference group; US, United States.

Within the mITT population, 73.8% (499/676) of participants achieved treatment success at 8 weeks (Figure 3). Similar treatment success rates were observed across most demographic subgroups including sex, race, ethnic group, site geography, and number of prior CDI episodes (Figure 4). Advanced age (<65 years, ≥65 years: odds ratio [OR], 1.54 [95% confidence interval {CI}, 1.09–2.18; *P* = .014) and CCI score ≥3 (<3, ≥3: OR, 1.51 [95% CI, 1.07–2.14]; *P* = .02) were associated with an increased risk of treatment failure. Of the 499 RBL responders, 91.0% remained CDI recurrence-free through 6 months (Figure 3). Across demographic subgroups, similar rates of sustained clinical response were observed (Figure 4). Among first-recurrence participants (n = 180), 70.6% had treatment success, of whom 89.0% remained CDI recurrence-free through 6 months.

### Second Course of RBL

Among 151 participants who experienced CDI recurrence within 8 weeks of RBL administration and met the definition of treatment failure, 121 elected to receive a second course of RBL within 21 days of failure. Antibiotics were not required prior to a second course of RBL and were administered at the discretion of the study investigator; however, most participants received antibiotics for CDI between treatment failure and a second RBL administration (vancomycin, n = 93 [76.8%]; fidaxomicin, n = 17 [14%]; metronidazole, n = 4 [3.3%]).

Within 8 weeks of second course administration, 38.8% of participants reported TEAEs (Supplementary Table 4). Between 8 weeks and 6 months of follow-up, 15.7% of participants reported TEAEs. Most TEAEs were mild-to-moderate gastrointestinal events, similar to the single course cohort. No participants experienced major complications of CDI following a second course of RBL.

Following a second course of RBL, 55.4% of participants achieved treatment success (Supplementary Table 5). In sum, 83.7% (566/676) of participants achieved treatment success with 1 or 2 courses of RBL. Of the 67 participants who achieved treatment success following a second course of RBL, 88.1% remained CDI recurrence free through 6 months.

## DISCUSSION

PUNCH CD3-OLS was the largest evaluation of safety and efficacy of a live microbiota-based therapy in a “real–world” patient population. Findings are reported for 697 participants who received RBL, including those with comorbid and mild-to-moderate immunocompromising conditions. Approximately half of participants were 65 years or older and one-quarter received RBL after experiencing their first CDI recurrence. The safety and efficacy of RBL in this study were largely consistent with outcomes of other studies in the clinical development program.

The primary objective of this study was to assess the safety of RBL, as many participants would have met exclusion criteria in earlier trials based on comorbidities. Most TEAEs reported in this study were mild or moderate in severity and transient. Serious TEAEs were most frequently related to CDI and preexisting conditions, regardless if participants received 1 or 2 courses of RBL. No clustering of terms or types of serious TEAEs was observed to signal safety concerns. Additionally, no reported serious infections were assessed as related to RBL. Overall, results are consistent with those from an integrated analysis of safety data from all studies in the RBL clinical trial program comprising 1061 participants [10]. Given the inclusion of higher-risk participants in this cohort, such as those with IBD and mild-to-moderate immunocompromising conditions, these data support safe use of RBL in complex populations. Additional analyses in these subgroups are forthcoming.

The 73.8% treatment success rate at 8 weeks and 91.0% sustained clinical response rate at 6 months reported here are comparable with results from the pivotal PUNCH CD3 RCT, which reported success rates of 70.6% and 92.1%, respectively [8]. The findings of the present study (PUNCH CD3-OLS) are more representative of real-world patients with complex comorbidities compared with PUNCH CD3, which excluded participants with IBD and immunocompromising conditions. As advanced age and comorbid conditions are known risk factors for rCDI [4, 5], it was expected that higher rates of treatment success would be observed in younger participants and those with fewer comorbidities. Univariate analyses confirmed that age and CCI score at screening were associated with risk of recurrence in PUNCH CD3-OLS. However, no differences among subgroups were observed on multivariate analysis.

Up to 65% of patients with a first recurrence are likely to experience a future CDI episode after antibiotic treatment, highlighting an unmet need for this population [11]. In the current study, treatment success rates among participants experiencing a first CDI recurrence were consistent with the broader study population (70.6% [127/180] vs 73.8% [368/492], respectively). Following a first rCDI, patient risk factors become very important and can make a patient high risk for rCDI. It has been suggested RBL should be considered as preventive therapy in high-risk patients after antibiotic treatment of their first CDI recurrence [12]. In a post hoc analysis of the pivotal phase 3 trial, the probability of treatment success at 8 weeks for participants experiencing first CDI recurrence was 81% for RBL recipients (n = 53) and 60% for placebo recipients (n = 33), representing a 21% absolute difference (crude proportions, 79.2% vs 60.6%; relative risk, 0.53; *P* = .06) [13]. RBL recipients experienced greater improvements in quality of life relative to placebo recipients using the *Clostridioides difficile* Health-related Quality-of-Life Questionnaire (Cdiff32). Findings collectively demonstrate the efficacy of RBL in preventing rCDI after first recurrence, which may increase clinicians’ confidence in the potential utility of live microbiota-based therapies at this stage of the rCDI treatment paradigm.

Some patients need multiple therapeutics to stop the cycle of rCDI. In this study, 55.4% of participants who received a second course of RBL within 3 weeks of their initial course experienced treatment success over the subsequent 8 weeks, and 88.1% of treatment responders experienced sustained clinical response to 6 months, consistent with treatment success rates in this subgroup of the pivotal phase 3 trial [8]. Most participants who received a second course of RBL in this study received rCDI treatment with antibiotics prior to administration. As per FDA labeling, SOC antibiotics are recommended to treat acute rCDI prior to the administration of RBL [9]; however, the study protocol did not specify this as a requirement, and therefore antibiotic retreatment trends in this study reflect clinician preference. While participant characteristics predictive of second course success are unknown, this strategy could benefit approximately half of those who do not experience initial treatment success, with sustained results. There were no safety concerns associated with 2 courses versus 1 course of RBL in this study.

These results should be interpreted with consideration. The single-arm open-label design may introduce bias due to the lack of blinding of participants and study administrators. Lack of a control group limits further evaluation of efficacy and safety. However, this study was the largest to date for any live microbiota-based therapy and findings were consistent with the pivotal phase 3 study. The study population and diagnostics herein reflect real-world scenarios, unlike most RCTs. Inclusion of participants using diagnostics commonly encountered in clinical practice can be considered a strength that minimizes selection bias favoring “ideal” rCDI study candidates. Concerns may arise over inclusion of participants with colonization versus true infection given that most rCDI diagnoses used PCR. Under that pretense, in theory, treatment success rates with RBL may otherwise be higher than observed here. Diagnoses were made by study investigators who have experience managing patients with rCDI, and participants were required to have CDI symptom (eg, diarrhea) resolution with SOC antibiotics. These variables likely mitigated risk of enrolling colonized participants. This trial did not report long-term data beyond 6 months; however, safety findings are consistent with previous studies in the RBL clinical trial program that had 2-year follow-up periods [13, 14]. Notably, univariate regression analysis focused on nonmodifiable patient characteristics that may have impacted safety and/or efficacy findings. The impact of modifiable factors was not studied. A previously conducted pooled analysis of participants administered a single course of RBL from PUNCH CD2 and PUNCH CD3 suggested that proton pump inhibitor (PPI) use and antibiotic washout period may impact efficacy outcomes. Greater treatment effect sizes were observed for participants not receiving a PPI or H2 receptor antagonist versus those who were, as well as for participants who received a 3-day antibiotic washout relative to 1–2 days [15]. While these factors were not specifically evaluated in this study, they may impact efficacy findings and may be helpful considerations during clinical decision making. Finally, the study population was predominantly White despite efforts to expand participant diversity, which potentially limits generalizability of findings to other racial groups.

## CONCLUSIONS

In conclusion, RBL was safe and efficacious in preventing rCDI among participants who were representative of those encountered in routine medical practice. These findings provide insight into the safety and efficacy of RBL in a large patient population inclusive of those usually excluded from prospective clinical trials. Preventing rCDI has the potential to reduce the high burden and costs associated with the cycle of recurrence for patients, clinicians, and healthcare systems.

## Supplementary Data

Supplementary materials are available at *Clinical Infectious Diseases* online. Consisting of data provided by the authors to benefit the reader, the posted materials are not copyedited and are the sole responsibility of the authors, so questions or comments should be addressed to the corresponding author.

## Supplementary Material

ciae437_Supplementary_Data

## Notes

***Author contributions.*** Conceptualization: L. B. Methodology: B. G., M. G., and L. B. Data curation: B. G. and M. G. Validation: All authors. Formal analysis: B. G. and M. G. Writing and reviewing: All authors.

***Acknowledgments.*** The authors thank all of the participants and their families and caregivers, as well as the investigators and site staff. Medical writing support, under the guidance of the authors, was provided by Rebecca Budgett, PhD (ApotheCom, Yardley, Pennsylvania) and was funded by Ferring Pharmaceuticals, Parsippany, New Jersey. Copy editing support was provided by Monique Bidell, PharmD, an employee of Ferring Pharmaceuticals.

***Data availability.*** Ferring will provide access to individual de-identified participant data upon request via a secure portal, to researchers whose proposals meet the research criteria and other conditions. To gain access, data requestors must enter into a data access agreement with Ferring.

***Ethics statement.*** PUNCH CD3-OLS was conducted across the US and Canada under a US FDA Investigational New Drug Application and a Health Canada Clinical Trial Application. Each participating site obtained approval of the protocol from the institutional review board/research ethics board. The study was conducted in accordance with the ethical standards of the 1964 Declaration of Helsinki. Participants provided written informed consent to participate prior to initiation of any study procedures.

***Disclaimer.*** Ferring Pharmaceuticals was involved in the study design, collection, analysis, and interpretation of the data; writing of the report; and the decision to submit the paper for publication.

***Financial support*.** This work was supported by Ferring Pharmaceuticals.
