# Supplementary material for: PUNCH CD3-OLS: A Phase 3 Prospective Observational Cohort Study to Evaluate the Safety and Efficacy of Fecal Microbiota, Live-jslm (REBYOTA) in Adults With Recurrent Clostridioides difficile Infection
Source: Clin Infect Dis. 2024 Aug 24;80(1):43–51. doi: 10.1093/cid/ciae437 (PMC11797394; doi:10.1093/cid/ciae437)
Supplement: ciae437_Supplementary_Data [file ciae437_supplementary_data.docx]

**Supplementary Methods**

**PUNCH CD3-OLS Full Inclusion and Exclusion Criteria**

| **Inclusion criteria** | **Exclusion criteria** |
| --- | --- |
| 1. Adults aged 18 years old or older 2. Medical record documentation of either a) a current diagnosis or history of recurrent CDI as determined by the treating physician, b) or had at least 2 episodes of severe CDI resulting in hospitalization 3. Was currently taking or was just prescribed SOC antibiotics to control CDI-related diarrhea at the time of enrollment. Note: CDI diarrhea must be controlled (<3 unformed/loose, ie, Bristol Stool Scale type 6-7, stools/day for the 2 consecutive days prior to the washout period) while taking antibiotics during screening. During the washout period (24 to 72 hours), SOC antibiotics were no longer taken to allow the participant to pass or “wash out” these treatments which may have an impact on RBL 4. Was willing and able to have an enema(s) 5. Was willing and able to complete the stool and blood testing required for the study 6. Agreed to not take non-dietary probiotics from Screening through 8 weeks after receiving the last study enema (including over the counter and prescription) 7. Agreed to not take any oral vancomycin, metronidazole, fidaxomicin, rifaximin, nitazoxanide, bezlotoxumab, or intravenous immunoglobin through the 8-week follow-up assessment unless newly prescribed by a treating investigator during the course of the study as a result of recurrent CDI diagnosis. Note: Use of intravenous immunoglobin for treatment of a non-CDI indication was allowed 8. Agreed to practice a form of effective contraception during study participation; did not apply to persons with documented non–child-bearing potential 9. Had a negative urine pregnancy test at the time of enrollment and on the day of each enema prior to administration (persons of child-bearing potential only) 10. Was willing and able to provide informed consent, and local privacy authorization as applicable 11. Was willing and able to complete the required Subject Diary 12. Was willing and able to meet all study requirements, including attending all assessment visits and telephone calls | 1. A known history of refractory CDI 2. Had continued CDI diarrhea despite being on a course of antibiotics prescribed for CDI treatment 3. Required systemic antibiotic therapy for a condition other than CDI 4. Previous participation in a Rebiotix clinical study receiving RBL 5. FMT within the past 6 months 6. FMT with an associated serious adverse event related to the FMT product or procedure 7. Received bezlotoxumab (CDI monoclonal antibodies) within the last year 8. Disease symptoms (diarrhea) caused by a confirmed intestinal pathogen other than *C. difficile* 9. Currently had a colostomy 10. Intraabdominal surgery within the last 60 days. Note: laparoscopic procedures that did not involve the gastrointestinal tract were permitted 11. Had planned surgery requiring perioperative antibiotics through the 8-week follow-up assessment 12. Life expectancy of <6 months 13. CD4 count <200/mm3 during screening 14. An absolute neutrophil count of <1000 cells/μL during screening 15. Had known or suspected current (<90 days) illicit drug use. Note: marijuana use was allowed 16. Was pregnant, breastfeeding, or intended to become pregnant during study participation 17. Participating in a clinical study of another investigational product (drug, device or other) and has not completed the required follow-up period 18. Was eligible for another RBL study 19. Individual, in the opinion of the investigator, for whatever reason, should be excluded from the study |

CDI, *Clostridioides difficile* infection; FMT, fecal microbiota transplant; RBL, fecal microbiota, live-jslm; SOC, standard-of-care.

**Serious TEAE Definition**

A serious treatment-emergent adverse event (TEAE) was defined as an adverse event or adverse reaction with an onset on or after the first day of administration and resulting in any of the following outcomes:

- Death
- Life-threatening event
- Hospitalization >24 h or prolongation of an existing hospitalization
- Persistent or significant incapacity or substantial disruption of the ability to conduct normal life functions
- Congenital anomaly/birth defect
- Important medical event

**Evaluation of Efficacy**

Efficacy assessments were conducted at each scheduled visit (weeks 1, 4, and 8 [treatment success], months 4 and 6 [sustained clinical response]). CDI recurrence (ie, treatment failure) was defined as the presence of CDI diarrhea within 8 weeks after RBL administration. Recurrence was confirmed using C. diff Quik Check Complete^®^ (enzyme immunoassay test for glutamate dehydrogenase [GDH] and toxins A and B) in a central laboratory to ensure consistency across trial sites. Polymerase chain reaction (PCR)-based testing was carried out following discordant results (eg, GDH-positive and toxin-negative) to confirm treatment failure. An independent adjudication committee assessed treatment outcome, providing the results used for reporting. An additional efficacy endpoint was treatment success 8 weeks after administration of a second course of RBL, if applicable.

**Multivariate Logistic Regression Analysis**

The sensitivity of treatment success at 8 weeks was analyzed using a multivariate logistic regression, adjusting for potential interactions with the following covariates: age (< 65 years, ≥ 65 years), sex (female, male), race group (White, Non-white), ethnicity (Hispanic-Latino, not Hispanic-Latino), site geography (outside the US, Eastern US, Southern US, Northern US, Western US), and the number of previous episodes of CDI at baseline (≤ 3, > 3). The model was forced to include the main effects of each demographic and baseline characteristic and then used a forward selection procedure to include pairwise interaction terms at a *p*-value threshold of 0.2. The resulting model contained interactions between age group and sex, age group and site geography, and sex and CCI category.

**Study Population Definitions**

Safety endpoints were assessed in the safety population, defined as all participants in which RBL administration was attempted or completed.

The analysis of treatment success and sustained clinical response was performed on the modified intent-to-treat population, defined as all participants who were successfully administered RBL but excluding those in whom administration was attempted but not completed and those who discontinued from the study prior to evaluation of treatment failure/success for the primary endpoint if the reason for exit was unrelated to CDI symptoms.

**Supplementary Table 1.** Participant Disposition (safety population)

| **n (%)** | **RBL (N = 697)** |
| --- | --- |
| Participants who reached week 1 | 690 (99.0) |
| Participants who reached week 4 | 676 (97.0) |
| Participants who reached week 8 | 657 (94.3) |
| Participants who reached month 4 | 645 (92.5) |
| Participants who reached month 6 | 636 (91.2) |
| Participants who completed the study | 635 (91.1) |
| Participants who discontinued the study | 62 (8.9) |
| Reasons for study discontinuation | |
| AE | 2 (0.3) |
| Death | 6 (0.9) |
| Failure to comply with study requirements | 3 (0.4) |
| Withdrawal by investigator | 3 (0.4) |
| Lost to follow-up | 22 (3.2) |
| Termination by study sponsor | 0 (0) |
| Withdrawal by participant | 25 (3.6) |
| Other | 1 (0.1) |
| Participants treated and withdrew prior to 8-week primary endpoint evaluation | 30 (4.3) |
| AE | 1 (0.1) |
| Death | 4 (0.6) |
| Failure to comply with study requirements | 2 (0.3) |
| Withdrawal by investigator | 1 (0.1) |
| Lost to follow-up | 11 (1.6) |
| Termination by study sponsor | 0 (0) |
| Withdrawal by participant | 11 (1.6) |
| Other | 0 (0) |

AE, adverse event; RBL, fecal microbiota, live-jslm.

**Supplementary Table 2.** Summary of TEAEs Following RBL Administration by Baseline Characteristic (safety population)

|  | **RBL (N = 697)** |
| --- | --- |
|  | **All TEAEs, events/participants (% of participants)** |
| Age Group | |
| <65 years (n = 359) | 588/212 (59.1) |
| ≥65 years (n = 338) | 537/186 (55.0) |
| Racial Group | |
| White (n = 654) | 1053/375 (57.3) |
| Non-White (n = 43) | 72/23 (53.5) |
| Sex | |
| Male (n = 210) | 278/111 (52.9) |
| Female (n = 487) | 847/287 (58.9) |

RBL, fecal microbiota, live-jslm; TEAE, treatment-emergent adverse event.

**Supplementary Table 3.** CDI-Related Complications Following RBL Administration (safety population)

|  | **RBL (N = 697)** |
| --- | --- |
| Participants with CDI-related complications, n (%)^a^ | 4 (0.6) |
| Complication, events/participants (% of participants)^b^ | |
| Death^c^ | 1/1 (0.1) |
| Septic shock | 6/4 (0.6) |
| Toxic megacolon | 0 |
| Colonic perforation | 0 |
| Emergency colectomy | 0 |
| ICU admission | 5/2 (0.3) |

CDI, *Clostridioides difficile* infection; ICU, intensive care unit; RBL, fecal microbiota, live-jslm.

^a^All CDI-related complications occurred within 8 weeks of first course of RBL.

^b^Complication categories are not mutually exclusive.

^c^Cause of death was sepsis (deemed unrelated to RBL).

**Supplementary Table 4.** Summary of TEAEs Within 8 Weeks and Between 8 Weeks and 6 Months of Follow-Up of Second Course of RBL (safety population)

|  | **Within 8 Weeks  (N = 121)** | **8 Weeks to 6 Months**  **(N = 121)** |
| --- | --- | --- |
|  | **Events/participants (% of participants)** | |
| All TEAEs | 105/47 (38.8) | 65/19 (15.7) |
| TEAEs by maximum severity^a^ | |  |
| Mild | 54/21 (17.4) | 26/5 (4.1) |
| Moderate | 45/21 (17.4) | 37/12 (9.9) |
| Severe | 6/5 (4.1) | 2/2 (1.7) |
| Potentially life-threatening | 0/0 (0.0) | 0/0 (0.0) |
| All serious TEAEs | 4/3 (2.5) | 3/3 (2.5) |
| Serious TEAEs by maximum severity^a^ | |  |
| Mild | 1/1 (0.8) | 0/0 (0.0) |
| Moderate | 0/0 (0.0) | 1/1 (0.8) |
| Severe | 2/2 (1.7) | 2/2 (1.7) |
| Potentially life-threatening | 0/0 (0.0) | 0/0 (0.0) |
| Serious TEAEs by relatedness^b^ | |  |
| Related to RBL | 0/0 (0.0) | 0/0 (0.0) |
| Related to administration procedure | 0/0 (0.0) | 0/0 (0.0) |
| Related to CDI | 3/2 (1.7) | 0/0 (0.0) |
| Related to preexisting conditions | 1/1 (0.8) | 1/1 (0.8) |
| TEAEs leading to withdrawal from study | 0/0 (0) | 0/0 (0.0) |
| TEAEs leading to death | 0/0 (0) | 0/0 (0.0) |

CDI, *Clostridioides difficile* infection; RBL, fecal microbiota, live-jslm; TEAE, treatment-emergent adverse event.

^a^Both participants and events are by maximum severity per participant.

^b^Relatedness categories are not mutually exclusive.

**Supplementary Table 5.** CDI Recurrence Rate for Participants Administered a Second Course of RBL (mITT population)

| **% (n)** | **RBL (N = 121)** |
| --- | --- |
| Treatment success^a^ | 55.4 (67) |
| Treatment failure | 24.0 (29) |
| Indeterminate^b^ | 20.7 (25) |

CDI, *Clostridioides difficile* infection; mITT, modified intent-to-treat; RBL, fecal microbiota, live-jslm.

^a^Treatment success was defined as the absence of CDI diarrhea through 8 weeks after the second course of RBL.

^b^The protocol-specified definition of treatment success or treatment failure was not met.
